# Complete Versus Incomplete Revascularization in Elderly Patients With Myocardial Infarction: A Systematic Review and Meta-Analysis

**DOI:** 10.7759/cureus.74068

**Published:** 2024-11-20

**Authors:** Cesar Intriago, Cristopher-Josué Escudero, Jesús Endara-Mina, Oscar E Dávila, Miriam J Zúñiga, Génesis D Loor, José L Villamarín-Corrales, Juan C Gaibor, Rafael López-Carrera, Luis S Loya, Angélica Lema

**Affiliations:** 1 Research, Larkin Community Hospital, South Miami, USA; 2 Research, Eugenio Espejo Specialties Hospital, Quito, ECU; 3 Teaching and Research, Calderón Teaching Hospital, Quito, ECU; 4 Research, Vozandes Hospital, Quito, ECU; 5 Interventional Cardiology, Metropolitan Hospital, Quito, ECU; 6 Vascular Surgery, Pablo Arturo Suarez General Hospital, Quito, ECU; 7 Research, Calderón Teaching Hospital, Quito, ECU; 8 Geriatrics, Geriatrix Clinic, Quito, ECU

**Keywords:** coronary artery disease, coronary revascularization, major adverse cardiovascular events, myocardial infarction, older adults

## Abstract

Coronary artery disease (CAD) is the leading cause of mortality in the United States, and percutaneous coronary intervention (PCI) is established as the standard after an acute episode of CAD. This review assessed the use of complete revascularization (CR) or incomplete revascularization (IR) in older adults, who present a higher cardiovascular risk. The aim is to define the effectiveness of both procedures in this population, focusing on major adverse cardiovascular events (MACE), myocardial infarction (MI), and all-cause mortality (ACM). A literature search identified 15 studies, evaluated using the Risk of Bias 2 (RoB 2) tool and the Risk of Bias in Non-Randomized Studies of Interventions I (ROBINS-I) tool for bias risk. Despite positive results in recent studies, this meta-analysis does not show the superiority of CR, demonstrating a lack of benefit in reducing mortality, myocardial infarction, and adverse events in the ≥ 70-year-old age group.

## Introduction and background

Coronary artery disease (CAD) remains the leading cause of global mortality and morbidity, accounting for approximately 16% of all deaths worldwide and resulting from a complex interplay of environmental, lifestyle, and genetic factors. According to the WHO (2020), CAD is responsible for about 9 million deaths annually, while the Global Burden of Disease Study (2021) estimates it affects 126 million people globally, representing 17.2% of the world population [1-3], and the standard treatment after an acute episode of CAD is percutaneous coronary intervention (PCI) [4]. In recent years, there has been extensive research on whether to perform PCI based on complete revascularization (CR), which treats all coronary arteries, or incomplete revascularization (IR), which only treats the affected artery. The balance between the two options has been well-defined, and CR is the most effective [5-7]. However, there is very little current evidence on the use of CR or IR in older adults, who are at a higher risk of developing major adverse cardiovascular events (MACE) in the future.

Older adults have several comorbidities that make them susceptible to developing more complications and, at the same time, a higher risk of nephropathy due to contrast use. This population suffers from coronary artery disease with a varied and more complicated anatomy [6-8].

According to studies, it is estimated that by the year 2050, the older adult population will double. It is well-known that the prevalence of CAD is proportional to advanced age [4,7]. We aim to define which of the two alternatives, CR or IR, is the most effective procedure in this age group. Therefore, we have planned this meta-analysis and systematic review in which we assess the use of CR versus IR in an older population with CAD, using mortality as the primary outcome and other secondary outcomes such as myocardial infarction (MI) and the need for a second revascularization.

Our objective was to determine the risk associated with MACE, all-cause mortality, and myocardial reinfarction related to CR versus IR in patients over 70 years old.

## Review

Methodology

This systematic review and meta-analysis were performed and reported by the Cochrane Collaboration Handbook for Systematic Reviews of Interventions and the Preferred Reporting Items for Systematic Reviews and Meta-Analysis (PRISMA Statement guidelines) [9,10]. This study is registered in PROSPERO with the identification CRD42023475630. The protocol was changed once in the PROSPERO system, cause the age of the population was updated according to the founded articles.

Eligibility Criteria

No restrictions were applied regarding the publication date, publication status, or language. The inclusion criteria encompassed the following: (1) randomized controlled trials (RCTs) and observational studies; (2) studies including patients over 70 years old; (3) studies with a control group; and (4) articles reporting on the risk associated with all-cause mortality, myocardial reinfarction, and the need for CR. Our exploration was specifically limited to studies involving human subjects. We excluded case reports, case series, letters, editorials, narrative reviews, systematic reviews, meta-analyses, and pre-prints from our scope.

Search Strategy and Data Extraction

We conducted systematic searches on PubMed, Scopus, and Web of Science. The search was performed on October 31, 2023, using the following search terms: "multivessel disease", "coronary artery disease", "myocardial infarction", "coronary occlusion," "Acute Coronary syndrome”, "myocardial Ischemia", "coronary angioplasty”, "percutaneous coronary intervention", "revascularization", “culprit”, "culprit-Only" and "complete revascularization”. The references of all included studies, previous systematic reviews, and meta-analyses were also manually searched to find additional studies. The search strategy was developed by two authors (C.I. and C.E.), with the terms and approach reviewed and refined by J.E. and J.V. prior to application. Duplicate documents were manually removed by O.D.

Screening of titles and abstracts was conducted independently by M.Z. and G.L., following pre-defined eligibility criteria. Any disagreements at this stage were resolved with R.L. as an adjudicating reviewer. For the full-text review, two paired groups were established (M.Z., G.L., O.D., and L.L.), with J.G. and A.L. acting as adjudicating reviewers. Articles that the paired reviewers could not categorize were directly assessed by the adjudicating reviewers. 

Quality Assessment

The quality assessment of studies included in the review was conducted by J.M., J.V., J.G., R.L., and A.L., who applied appropriate tools depending on the study design. For randomized studies, the authors used the Cochrane Collaboration’s Risk of Bias 2 (RoB 2) tool, which assesses bias across several domains. These domains include the randomization process, evaluating the adequacy of participant assignment to experimental and control groups; allocation concealment, determining whether treatment assignment was hidden to prevent biases; participant and personnel blinding, assessing whether participants and intervention personnel were aware of the assigned treatment; outcome assessor blinding, evaluating the awareness of assessors regarding treatment assignments; data integrity, covering loss to follow-up and missing data; and reporting bias, examining selective result reporting based on statistical significance or outcome direction. Each of these domains was evaluated qualitatively, with bias levels classified as low, high, or uncertain, rather than relying on numerical stratification.

On the flip side, the Risk of Bias in Non-Randomized Studies of Interventions I (ROBINS-I) tool, is focused on assessing bias risk (RoB) in outcomes from non-randomized studies of the effects of interventions (NRSIs) that analyze the health impacts of two or more interventions. While it draws inspiration from the Cochrane RoB tool designed for randomized trials, ROBINS-I operates within slightly different parameters. It encompasses seven domains aimed at identifying potential bias in NRSIs. The initial two domains tackle issues preceding the commencement of the interventions under comparison. The third domain delves into the categorization of the interventions themselves. The remaining four domains address concerns arising post-intervention initiation, as randomization does not shield against biases emerging after this stage.

Statistical Analysis

This systematic review and meta-analysis were conducted following the guidelines of the Cochrane Collaboration and the Preferred Reporting Items for Systematic Reviews and Meta-Analyses (PRISMA) statement. Hazard ratios (HR) with 95% confidence intervals (CI) were used to compare treatment effects for categorical endpoints. Cochran's Q test and I2 statistics were used to assess heterogeneity; P values less than 0.10 and I2>25% were considered significant for heterogeneity. A fixed-effects model was used for outcomes with low heterogeneity (I2 < 25%). Otherwise, a random-effects model by DerSimonian and Laird was employed. J.E. led the statistical analysis, collaborating with J.V. for quantitative interpretation. A sensitivity analysis using the generic inverse variance method was also conducted with adjusted risk estimates from observational studies, when available.

Ethical Declaration

In this systematic review, while ethics committee approval was not required as the study relied on secondary data sources, it is essential to ensure that all included studies adhere to the ethical principles outlined in the Declaration of Helsinki, particularly when involving an elderly population. Therefore, only studies with ethics committee approval were included.

Results

**Figure 1:**
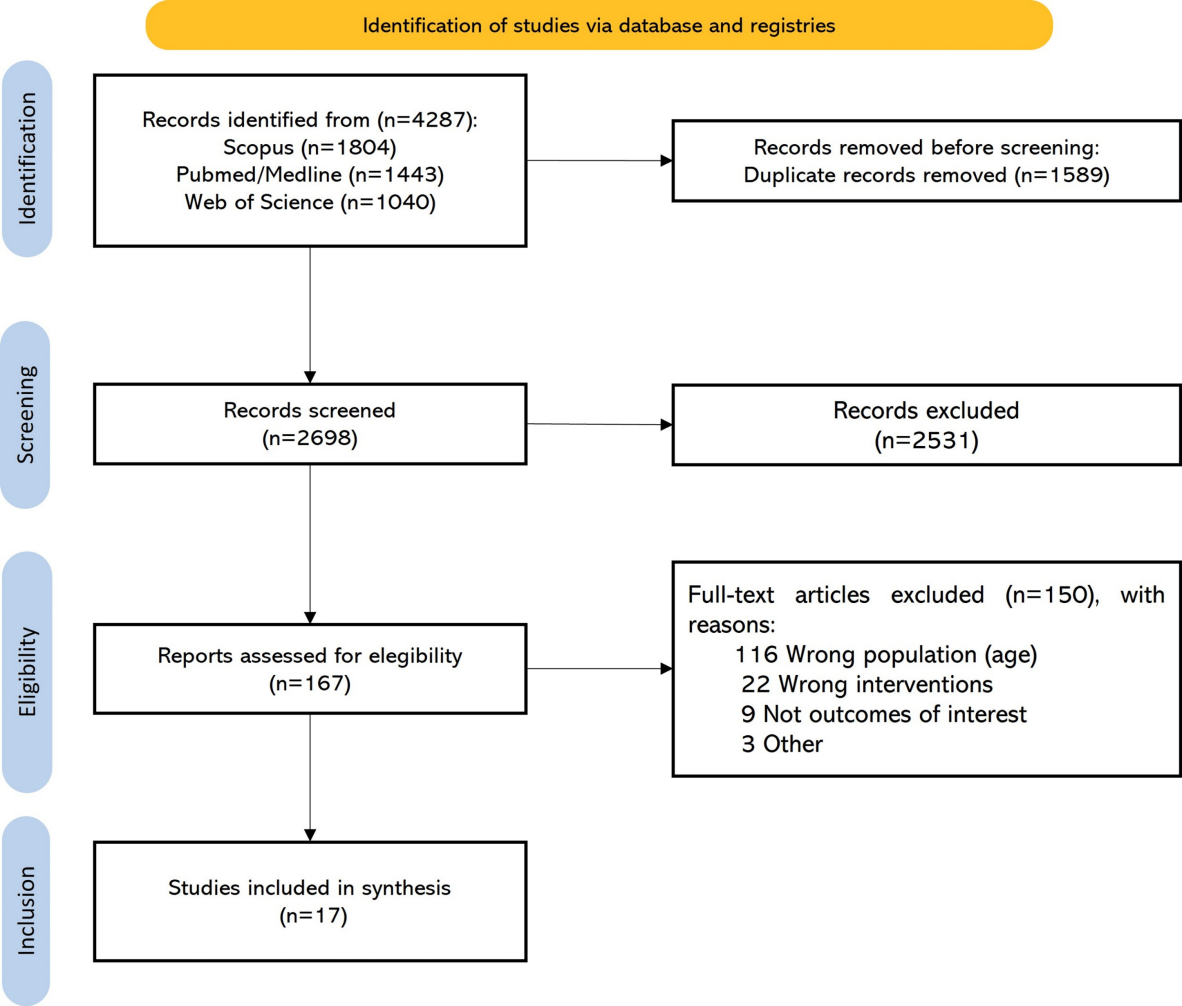
PRISMA flow diagram of study screening and selection. PRISMA: Preferred Reporting Items for Systematic Reviews and Meta-Analysis.

The results of the literature search and the selection of included studies are summarized in Figure 1. The initial database search identified a total of 4,285 publications. After excluding duplicates, 2,696 studies remained for the title and abstract screening. A full-text selection was performed on 165 studies, and ultimately, 17 studies were included in the systematic review and 13 articles were included in the metanalysis. Most studies were excluded due to having a population ineligible by age, and other reasons are detailed in Figure 1.

Characteristics of Studies

Our meta-analysis included 49,556 patients and the mean duration of follow-up was 30 months. As shown in Table 1, the base characteristics of the seventeen studies that we included were displayed. Of these, two are RCTs, seven are prospective studies, and eight are retrospective studies.

**Table 1:** Characteristics of the studies included in the systematic review. ^a^Mean months of follow-up, 12-44 months was the range of follow-up; *Population was taken from the study's total participants. Subgroup population was not reported. RCT: randomized controlled trial; CR: complete revascularization

Author(s)	Year	Study period	Type	Definition of CR used	Follow-up (months)	Number of patients
Biscaglia S, et al. [5]	2023	2019-2012	RCT	No definition	12	1445
Lu Y, et al. [6]	2023	2011-2019	Retrospective	Anatomic	36	790
Berezhnoi K, et al. [7]	2019	2014-2017	Prospective	Diameter	12	305
Marino M, et al. [8]	2022	2009-2010	Retrospective	Scored-Based	24	166
Kato T, et al [11]	2023	2016-2021	Prospective	Scored-based	60	39
Hwang D, et al. [12]	2019	2008-2014	Prospective	Scored-based	36	2863
Harada M, et al. [13]	2016	2013-2014	Prospective	Diameter	12	322
Iqbal M, et al. [14]	2021	2008-2015	Prospective	Anatomic	60	*8436
Hannan E, et al. [15]	2009	2003-2004	Retrospective	Anatomic	18	*11294
Bainey K, et al. [16]	2020	2007-2013	Retrospective	Scored-based	64	^*^9094
Wu C, et al. [17]	2013	2003-2005	Retrospective	Anatomic	60	8750
Rumiz E, et al. [18]	2018	2009-2015	Prospective	No definition	22	111
Magalhaes M, et al. [19]	2015	2003-2013	Retrospective	Diameter	12	*2132
Chen J, et al. [20]	2012	2005-2010	Prospective	Diameter	78	502
Song Y, et al. [21]	2011	2003-2006	Retrospective	Anatomic	35	269
Wang G, et al. [22]	2023	2003-2014	Retrospective	Diameter	12	1263
Joshi F, et al [23]	2020	2018-2022	RCT	Diameter	27^a^	110

Risk of Bias

The assessment of the risk of bias in randomized studies with the RoB-II tool is summarized in Table 2. The evaluation was carried out based on five domains, concluding that the risk of bias is low due to the size of the sample and the information available from the follow-up of practically all participants in the analyzed study.

**Table 2:** Analysis of risk of bias in the randomized studies included with RoB-II. RoB-II: risk of bias II.

Study	Bias from randomization process	Bias due to intended interventions	Bias due to missing outcome data	Bias in measurement of the outcomes	Bias in selection of the reported result	Overall risk of bias
Biscaglia S, et al. [5]	Low	Low	Low	Low	Low	Low
Joshi FR, et al. [23]	Low	Low	Low	Low	Low	Low

The assessment of the risk of bias in non-randomized studies with ROBINS-I tool is summarized in Table 3. The included studies represent a population with at least one major adverse cardiovascular event in an elderly population, and all studies included in the quantitative synthesis fulfilled this premise. Internally, seven biases were assessed, as described in Table 2. Confounding bias was reported in all studies with a moderate degree; however, considering the sample size and age grouping, it is not considered to affect the quality of the results.

**Table 3:** Analysis of risk of bias in the non-randomized studies included with ROBINS-I. ROBINS-I: Risk of bias in non-randomized studies of interventions I.

Study	Bias due to confounding	Bias in selection of participants into the study	Bias in classification of interventions	Bias due to deviations from intended interventions	Bias due to missing data	Bias in measurement of outcomes	Bias in selection of the reported result	Overall bias
Lu Y, et al. [6]	Moderate	Low	Moderate	Low	Low	Low	Low	Moderate
Bereznhoi K, et al. [7]	Moderate	Low	Low	Low	Low	Low	Low	Moderate
Marino M, et al. [8]	Moderate	Low	Low	Low	Low	Low	Low	Moderate
Kato T, et al. [11]	Moderate	Low	Low	Low	Low	Low	Low	Moderate
Hwang D, et al. [12]	Moderate	Low	Moderate	Low	Low	Low	Low	Moderate
Iqbal M, et al. [14]	Moderate	Low	Low	Low	Moderate	Low	Low	Moderate
Hannan EL, et al. [15]	Moderate	Low	Moderate	Low	Low	Low	Low	Moderate
Wu C, et al. [17]	Moderate	Low	Low	Low	Low	Low	Low	Moderate
Rumiz E, et al. [18]	Moderate	Low	Low	Low	Low	Low	Low	Moderate
Magalhaes M, et al. [19]	Moderate	Low	Low	Low	Low	Low	Low	Moderate
Song Y, et al. [21]	Moderate	Low	Low	Low	Moderate	Low	Low	Moderate
Wang G, et al. [22]	Moderate	Low	Low	Low	Moderate	Low	Low	Moderate

Qualitative Description

In the observational study by Kato T, et al. (2023), revascularization was performed in patients with CAD who also had peripheral artery disease (PAD) [11]. They found that CR was not significantly associated with a lower risk of MACE among patients > 75 years HR: 0.66; 95% CI: 0.35-1.24; p=0.20 [11].

In the observational study by Hwang D, et al. (2019), revascularization was performed in patients with CAD who also had chronic kidney disease (CKD) [12]. The study found that CR in patients > 70 years led to better clinical outcomes in patients with CKD than IR HR: 0.69; 95% CI: 0.53-0.89; p=0.005 [12].

In the observational study by Harada M, et al. (2016), revascularization was performed in elderly patients with CAD. They performed revascularization in 322 elderly patients with multi-vessel CAD [13]. A total of 165 (51.2%) received CR, and 157 (48.8%) received IR. They found that the incidence of MACE by survival analysis was significantly lower in the CR group than in the IR group (7.4% vs. 21.1%, p 0.001) [13].

In the observational study by Lu Y, et al. (2023), revascularization was performed in 1,018 elderly patients with CAD; 496 (48.7%) underwent CR and 522 (51.3%) received IR [6]. They followed up with the patients for three years. They found that CR was significantly associated with lower MACE risk than IR, HR: 0.65; 95% CI: 0.47-0.88; p=0.006. CR was also associated with a lower risk of cardiac mortality than IR, HR: 0.30; 95% CI: 0.12-0.75; p=0.01. The study found that CR demonstrates superior long-term outcomes than IR [6].

In the observational cohort study by Iqbal M, et al. (2021), consecutive patients were treated with percutaneous coronary intervention (PCI) between 2008 and 2015 [14]. The study found in a subgroup analysis that CR was more likely to confer survival in older patients’ group over 80 years, male patients, absence of renal disease, greater angina (CCS Class III-IV) and heart failure (NYHA Class III-IV) symptoms, and greater burden of CAD. CR was associated with higher survival in older adults over 80 years compared to IR, HR: 0,76; 95% CI: 0.59-0.98; p=0.039 [14].

The study by Berezhnoi K, et al. (2018), showed that the MACE rate in patients undergoing CR was significantly lower during the 12-month observation period [7]. They performed revascularization in 322 elderly patients over 80 years of age with multi-vessel CAD. 131 received CR, and 174 (48.8%) received IR. They found that the incidence of MACE was significantly lower in the CR group than in the IR group (14.5% vs. 27%, p=0.008). The effect of the independent variable of age on 1-year incidence of MACE was not statistically significant, HR: 1.02; 95% CI: 0.94-1.09; p=0.59 [7].

The observational study by Hannan E, et al. (2009) found in a subgroup analysis that patients aged 80 years or older with IR had borderline higher rates of MACE than those with CR, HR: 1.32; 95% CI: 1.00-1.75; p=0.05 [15]. There were 11.2 events in the CR group and 14.5 in the IR group. The study showed that patients undergoing coronary stenting who receive IR experience more adverse outcomes [15].

The study by Bainey K, et al. (2020) was an observational prospective study from the registry (April 1, 2007, to March 31, 2013) [16]. The study found in a subgroup analysis of 1,849 patients over 80 years that the cumulative events rate within the five-year follow-up of the study was not statistically significant between the complete and IR. The total number of events in the CR group was 26.7 vs 36.3 in the IR group [16].

The study by Wu C, et al. (2013) was based on observation and comparison, utilizing the Percutaneous Coronary Intervention Reporting System (PCIRS) registry in New York [17]. The PCIRS is a register containing information on every PCI in New York. They follow up on the mortality of patients for five years after a PCI with CR or IR. In a subgroup of people older than 80, the HR for mortality for IR versus CR was HR: 1.18; 95% CI: 0.99-1.41; p=0.07 in favor of IR. The number of deaths per case was 234/744 in the CR group vs 276/801 in the IR group. The study showed that IR was not statistically significantly associated with increased mortality vs CR [17].

The study by Rumiz E, et al. (2018), an observational prospective, single-center study, found no differences, neither in MACE nor in all-cause mortality, between CR and IR strategies in the > 75 years subgroup (39.1% vs. 48.2%, p=0.89) and (21.3% vs. 16.2%, p=0.59), respectively [18]. However, in the subgroup of patients <75 years, those with IR had a higher incidence of MACE as compared to CR patients (23.9% vs. 10.2%, log-rank p 0.024) [18]. Based on their findings, the routine CR strategy seems to be the best therapeutic option in younger patients. In contrast, the elderly population may not confer a clear clinical benefit during a long-term follow-up [18].

The study by Magalhaes M, et al. (2015) was a retrospective study of 2,132 consecutive patients with multivessel disease who underwent PCI at a single center between 2003 and 2013 [19]. The average age of the participants was 65 in the CR group and 67 in the IR group. The primary outcome was death or Q-wave myocardial infarction. The study found that CR in selected patients gives better outcomes than IR in multivessel CAD at one year of follow-up. The study also found that older age is an independent risk factor. The subgroup of age over 80 showed a HR: 5.64; 95% CI: 1.67-19.07; p=0.003 [19].

The study by Marino M, et al. (2022) found that in patients aged ≥85 years, a CR is associated with a better prognosis, especially in terms of nonfatal events [8]. The study included consecutive patients aged ≥85 years, presenting with acute coronary syndrome (ACS) and showing multivessel coronary disease at coronary angiography. The primary endpoint was the rate of major adverse cardiovascular events (MACEs, the composite of cardiovascular death, re-myocardial infarction, clinically driven percutaneous coronary intervention, and rehospitalization because of cardiac disease) at two years follow-up. They found that the incidence of MACE was significantly lower in the CR group than in the IR group (14.5% vs. 27%, p=0.008). The age variable was not a predictor of MACE at the two-year follow-up, HR: 0.96; 95% CI: 0.88-1.05; p=0.608 [8].

In the study by Chen J, et al. (2012), they prospectively screened patients aged ≥ 75 at the center (Institute of Geriatric Cardiology, Chinese PLA General Hospital) presenting with ACS from January 2005 to December 2010 [20]. A total of 502 patients were included; 230 patients obtained CR, and the other 272 patients underwent IR. They found that PCI characteristics and complications were similar between the CR and IR groups. The results showed no significant difference in cumulative survival rate (log-rank p = 0.051) [20].

In the study by Biscaglia S, et al. (2023), a prospective, multicenter, randomized controlled trial (Fire trial), they found that among patients who were 75 years of age or older, CR had a lower risk of a composite of death, MI, stroke, or ischemia-driven revascularization at one year [5]. A total of 1,445 underwent randomization (720 to CR and 725 to culprit-only revascularization). The primary outcome of the trial was death, MI, stroke, or any revascularization at one year. A primary-outcome event occurred in 113 patients (15.7%) in the CR group and 152 (21.0%) in the culprit-only group HR: 0.73; 95% CI: 0.57-0.93; p= 0.01 [5].

The study by Song Y, et al. (2011), an observational study, compared MACE (death, MI, or any revascularization) in 873 patients and 255 pairs generated by propensity-score matching [21]. CR was performed in 427 patients (48.9%) and IR in 446 (51.1%). The study performed a subgroup analysis in which the study found in a subgroup analysis of 269 patients over 70 years that CR was associated significantly with a lower risk of MACE, HR: 0.44; 95% CI: 0.25-077; p=0.46 in favor of CR [21].

The study by Wang G, et al. (2023), a retrospective observational study, was conducted using data obtained from the BleeMACS registry, comparing 1,263 patients evaluated from 2003 to 2014, and 445 of them received CR (35.2%) [22]. During the one-year follow-up period, CR was associated with a significantly decreased risk of MACE (13.7% vs. 20.5%, HR: 0.63, 95% CI: 0.45-0.88, p=0.007) and a lower risk of MI (5.9% vs. 9.9%, HR: 0.55, 95% CI: 0.33-0.92, p=0.02). However, it was not linked to a lower risk of all-cause death (9.5% vs. 13.5%, HR: 0.68, 95% CI: 0.45-1.02, p=0.06) [22].

Finally, the study by Joshi F, et al., using the database from the DANAMI-3 trial program performed from 2018 to 2022, a retrospective observational study was conducted, comparing 627 randomized patients to evaluate a primary endpoint composite of ACM, nonfatal reinfarction, and ischemia-driven revascularization of lesions in non-infarct-related arteries, with a median follow-up of 27 (range 12-44) months [23]. No significant statistical association was found with the primary outcome (15% vs. 22%, HR: 1.49, 95% CI: 0.57- 4.65, p=0.19). For secondary endpoints, such as ACM (4% vs. 7%, HR: 1.94, 95% CI: 0.57 - 6.63, p=0.29), MI (3% vs. 4%, HR: 1.23, 95% CI: 0.17- 8.71, p=0.80), and ischemia-driven revascularization (5% vs. 4%, HR: 0.59, 95% CI: 0.11 - 3.24, p=0.50), nonsignificant results were found [23].

Quantitative Synthesis

**Figure 2:**
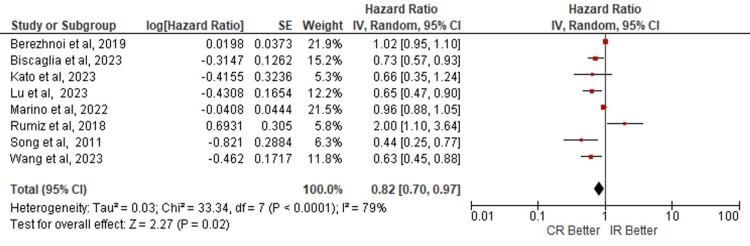
Risk of MACE in complete vs incomplete revascularization in elderly patients. Number of participants per group: Berezhnoi K, et al. [7], n=305; Biscaglia S, et al. [5], n=1445; Kato T, et al. [11], n=39; Lu Y, et al. [6], n=790; Marino M, et al. [8], n=166; Rumiz E, et al. [18], n=111; Song Y, et al. [21], n=269; Wang G, et al. [22], n=1263. CR: complete revascularization; IR: incomplete revascularization; SE: standard error; IV: inverse variance; CI: confidence interval; df: degrees of freedom; I²: heterogeneity (I-squared); Tau²: between-study variance (Tau-squared); Chi²: Chi-squared test; Z: Z-score for overall effect; P-value: statistical significance; MACE: major adverse cardiovascular events.

**Figure 3:**
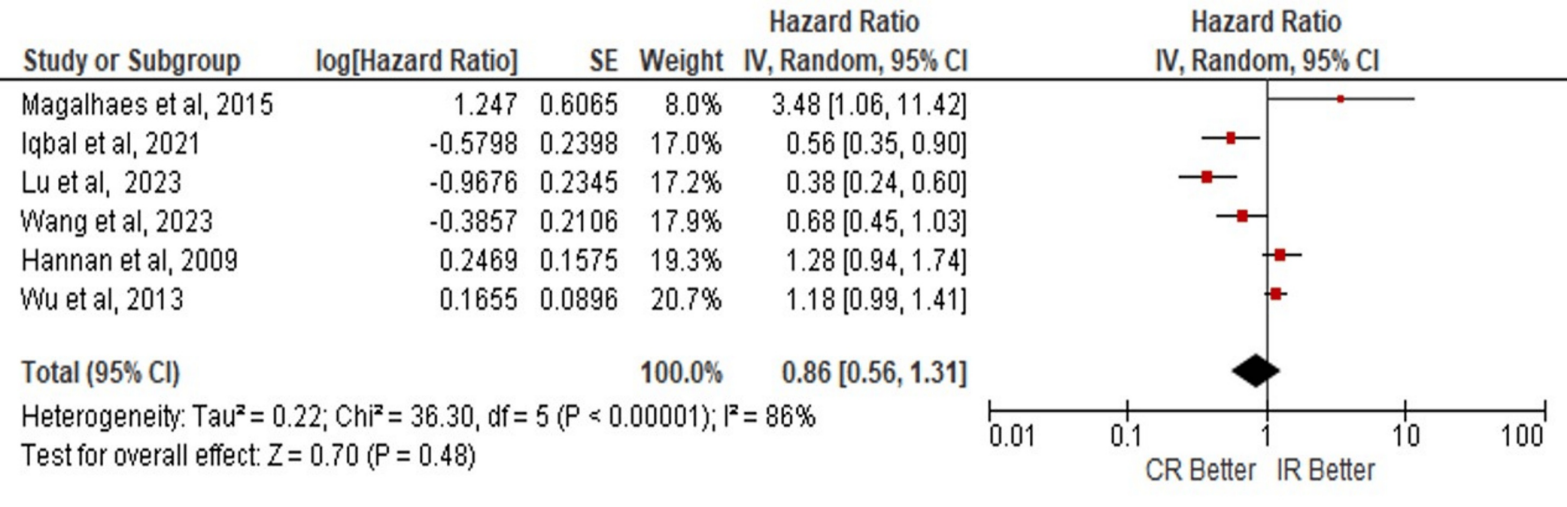
Risk of all-cause mortality in complete vs incomplete revascularization in elderly patients. Number of participants per group: Magalhaes M, et al. [19], n=2132; Iqbal M, et al. [14], n=8436; Lu Y, et al. [6], n=790; Wang G, et al. [22], n=1263; Hannan E, et al. [15], n=11294; Wu C, et al. [17], n=8750. CR: complete revascularization; IR: incomplete revascularization; SE: standard error; IV: inverse variance; CI: confidence interval; df: degrees of freedom; I²: heterogeneity (I-squared); Tau²: between-study variance (Tau-squared); Chi²: Chi-squared test; Z: Z-score for overall effect; P-value: statistical significance.

**Figure 4:**
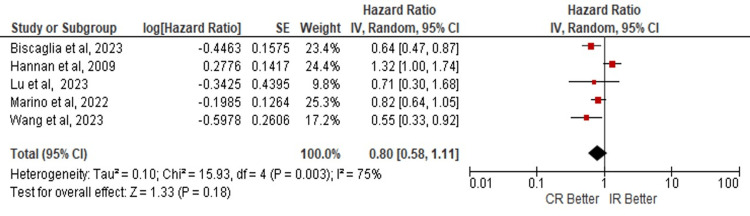
Risk of myocardial infarction in complete vs incomplete revascularization in elderly patients. Number of participants per group: Biscaglia S, et al. [5], n=1445; Hannan E, et al. [15], n=11294; Lu Y, et al. [6], n=790; Marino M, et al. [8], n=166; Wang G, et al. [22], n=1263. CR: complete revascularization; IR: incomplete revascularization; SE: standard error; IV: inverse variance; CI: confidence interval; df: degrees of freedom; I²: heterogeneity (I-squared); Tau²: between-study variance (Tau-squared); Chi²: Chi-squared test; Z: Z-score for overall effect; P-value: statistical significance.

The data analysis doesn't show statistical significance in the risk of developing MACE in these populations (HR: 0.82; 95% CI: 0.70 - 0.97; p=0.02) (Figure 2). For ACM, the mortality risk isn't significant for any of both procedure techniques (HR: 0.86; 95% CI: 0.56 - 1.31; p=0.48) (Figure 3); as well as the risk of MI shows no benefit with any of CR or IR, (HR: 0.80; 95% CI: 0.58 - 1.11; p=0.18) (Figure 4). The analyses were conducted using a random-effects model due to heterogeneity.

Subgroup Analysis

**Figure 5:**
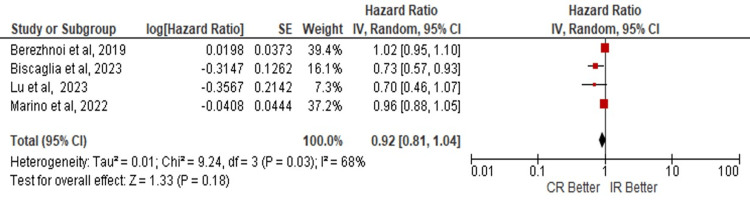
Risk of MACE in complete vs incomplete revascularization in the subgroup of elderly patients > 80 years old. Number of participants per group: Berezhnoi K, et al. [7], n=305; Biscaglia S, et al. [5], n=1445; Lu Y, et al. [6], n=790; Marino M, et al. [8], n=166. CR: complete revascularization; IR: incomplete revascularization; SE: standard error; IV: inverse variance; CI: confidence interval; df: degrees of freedom; I²: heterogeneity (I-squared); Tau²: between-study variance (Tau-squared); Chi²: Chi-squared test; Z: Z-score for overall effect; P-value: statistical significance, MACE: major adverse cardiovascular events.

**Figure 6:**
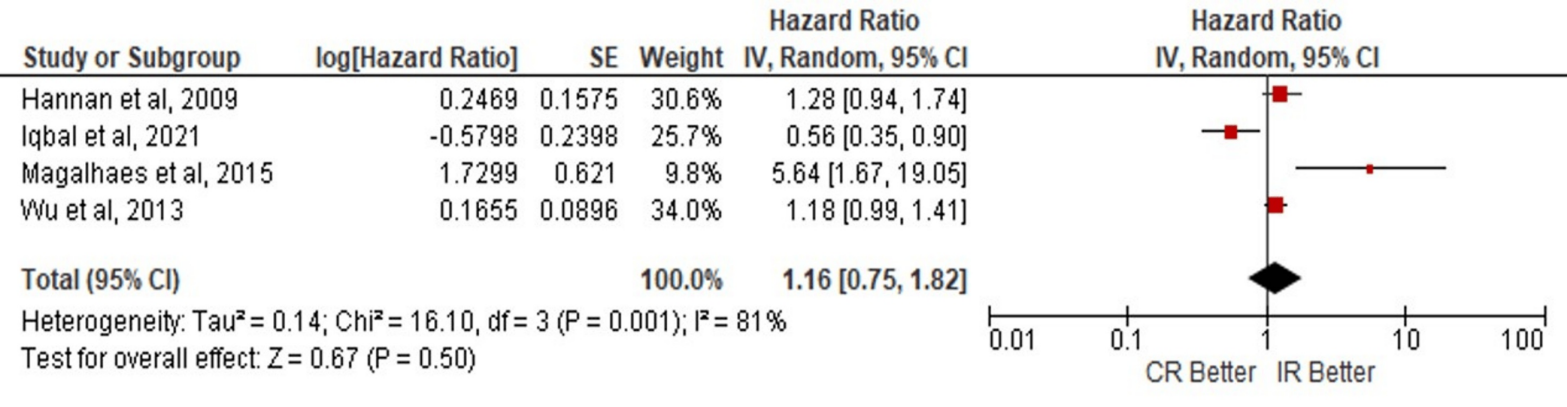
Risk of all-cause of mortality in complete vs incomplete revascularization in the subgroup of elderly patients> 80 years old. Number of participants per group: Hannan E, et al. [15]​​​​​​, n=11294; Lu Y, et al. [6], n=790; Iqbal M, et al. [14], n=8436; Magalhaes M, et al. [19], n=2132; Wu C, et al. [17], n=8750. CR: complete revascularization; IR: incomplete revascularization; SE: standard error; IV: inverse variance; CI: confidence interval; df: degrees of freedom; I²: heterogeneity (I-squared); Tau²: between-study variance (Tau-squared); Chi²: Chi-squared test; Z: Z-score for overall effect; P-value: statistical significance.

**Figure 7:**
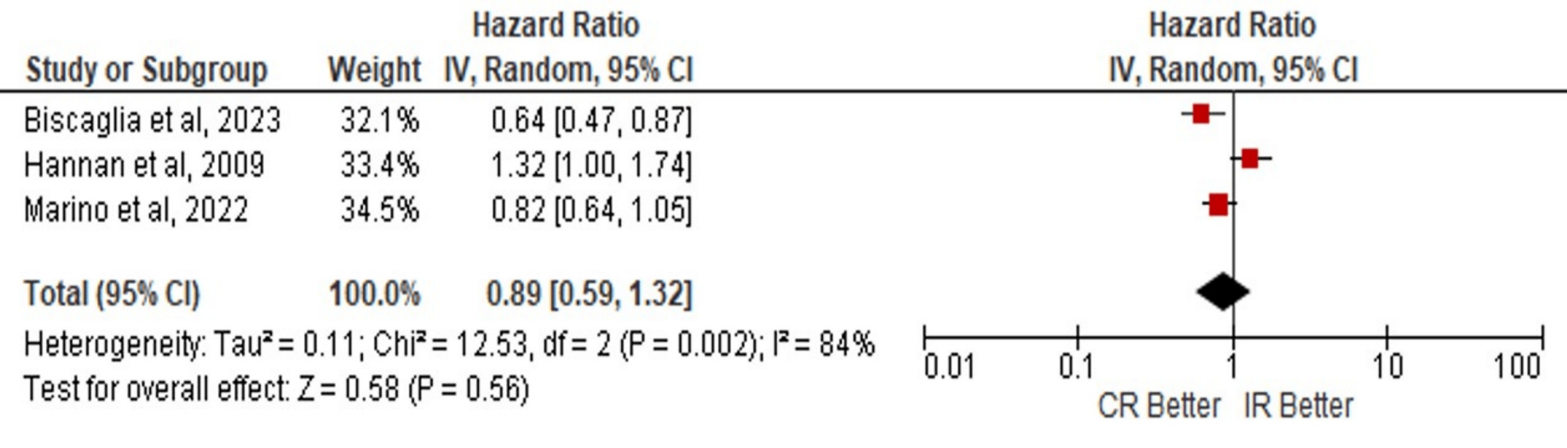
Risk of myocardial infarction in complete vs incomplete revascularization in the subgroup of elderly patients > 80 years old. Number of participants per group: Biscaglia S, et al. [5], n=1445; Hannan E, et al. [15], n=11294; Marino M, et al. [8], n=166. CR: complete revascularization; IR: incomplete revascularization; SE: standard error; IV: inverse variance; CI: confidence interval; df: degrees of freedom; I²: heterogeneity (I-squared); Tau²: between-study variance (Tau-squared); Chi²: Chi-squared test; Z: Z-score for overall effect; P-value: statistical significance.

The outcomes were analyzed in patients over 80 years of age where concerning the development of those MACE no statistically significant evidence was found that favors a CR or IR (HR: 0.92; 95% CI: 0.81 - 1.04; p=0.18) (Figure 5). No evidence was found that favors any of the revascularization techniques to the development of ACM (HR: 1.16; 95% CI: 0.75 - 1.82; p=0.50) (Figure 6) or MI (HR: 0.89; 95% CI 0.59 - 1.32; p=0.56) (Figure 7).

Discussion

CAD constitutes a significant cause of morbidity and mortality in older adult patients. Nevertheless, cardiovascular clinical trials have tended to underrepresent this population, even though older individuals are more prone than their younger counterparts to exhibit CAD of the left main trunk, multivessel CAD, and left ventricular dysfunction [24-26].

In the context of this systematic review and meta-analysis, a comparison was conducted between CR and IR after an MI. The main findings reveal (1) a small superiority of complete revascularization; (2) benefit in the population aged ≥70 years by preventing the occurrence of MACE; and (3) a clear lack of benefit in the age subgroup ≥80 years.

Recent randomized controlled trials such as COMPLETE (Complete vs Culprit-only Revascularization to Treat Multi-vessel Disease After Early PCI for STEMI) and CvLPRIT (Complete versus Lesion-only Primary PCI trial) demonstrate a positive association with CR [27,28]. In patients aged 75 years or older, the results from the BleeMACS study, involving 1,263 evaluated patients, showed that CR was associated with a significant decrease in the risk of MACE and a lower risk of myocardial infarction. However, it was not associated with a reduced risk of all-cause mortality (9.5% versus 13.5%, p=0.06) [22].

Similarly, several studies, including those by Biscaglia S, et al. (2023) [5], Berezhnoi K, et al. (2018) [7], Marino M, et al. (2022) [8], Hwang D, et al. (2019) [12], Harada M, et al. (2016) [13], Lu Y, et al. (2023) [6], Iqbal M, et al. (2021) [14], Hannan E, et al. (2009) [15] and Magalhaes M, et al. (2015) [19] attributed the benefit of experiencing fewer complications to the use of CR in elderly patients. However, studies conducted by Kato T, et al. (2023) [11], Wu C, et al. (2013) [17], and Chen J, et al. (2012) [20] did not show a statistically significant difference in the development of any revascularization techniques. Similarly, Rumiz E, et al. (2018) could not draw conclusive results for the adult population, stating that these cannot be fully extrapolated to the elderly population [18].

In contrast, the CRUSADE study emphasizes the significance of increasing age as a determining factor [29]. This analysis, based on a sample of individuals aged 90 or older, indicates a lower prevalence of revascularization use in this group compared to the population aged 75 to 89, with rates of 12.6% versus 40.1% (p < 0.001). Simultaneously, hospital outcomes indicate a higher risk of mortality (12.0% versus 7.8%) and adverse events (26.8% versus 21.3%) [29]. In another study focused on octogenarians and nonagenarians who underwent percutaneous intervention, mortality rates were observed at 30 days (17.2% versus 25.8%, p=0.028), one year (27.6% versus 32.5%, p=0.18), and five years (53.6% versus 57.3%, p=0.087), respectively. These findings consolidate the evidence that age plays a crucial role in clinical outcomes [30], supporting the conclusions of the CRUSADE study.

In another meta-analysis involving 89,883 patients regarding IR, CR was associated with lower long-term mortality, risk ratio (RR): 0.71, 95% CI: 0.65 to 0.77; p < 0.001, MI RR: 0.78, 95% CI: 0.68 to 0.90; p=0.001, and recurrent coronary revascularization RR: 0.74, 95% CI: 0.65 to 0.83; p < 0.001. These associations were observed in a population with a mean age ranging from 54 to 82 [31].

Similarly, other studies advocate for the use of CR in older individuals, even considering the presence of comorbidities as a factor for success [32]. It is evident that stronger evidence links CR to a decrease in the risk of mortality and MACE, particularly the latter when contrasted with our findings. However, these observations regarding CR are significantly more limited in elderly patients, as comorbidities gradually diminish the associated benefits of revascularization [33-35].

Limitations

A significant limitation was identified in the availability of studies focused on the elderly population in the context of comparing CR versus IR following a myocardial infarction. The scarcity of specific data for this demographic group affects the generalization of results and the ability to conduct thorough subgroup analyses.

Moreover, existing data lack a universally accepted and standardized definition of what constitutes an IR procedure. Gaba P, et al. [36] proposed a definition based on coronary angiography and fractional flow reserve (FFR) data. The suggested definition includes the inability to address all coronary segments with a stenosis diameter of ≥50% to 70% and an FFR ≤0.80, or stenosis >70% without FFR in vessels supplying a significant amount of viable myocardium. The lack of uniformity in the definitions used in the included studies may have introduced variability and bias into the results of the meta-analysis.

## Conclusions

CR appears to offer advantages in patients over 70 years old, specifically in the reduction of MACE, compared to IR. However, for other clinical outcomes, such as the risk of cardiovascular complications or long-term mortality, no statistically significant differences were observed between CR and IR in this older population. This suggests that, in terms of overall cardiovascular risk reduction, both techniques may be equally viable in patients over 70 years of age, with no clear advantage in terms of longevity.

Evidence from recent trials, such as the COMPLETE and CvLPRIT studies, supports the benefit of CR in younger populations, and the BleeMACS study has shown that CR significantly reduces the risk of MACE in patients over 75 years. Nonetheless, this benefit decreases in patients aged 80 and above, and studies in nonagenarians indicate that the benefit of CR becomes progressively less evident with advancing age due to comorbidity factors. This underscores the need to individualize clinical decisions in elderly patients, where balancing the benefits of CR against age-associated risks and comorbidities becomes crucial.
